# Retraction: Hyperthermia-Induced NDRG2 Upregulation Inhibits the Invasion of Human Hepatocellular Carcinoma via Suppressing ERK1/2 Signaling Pathway

**DOI:** 10.1371/journal.pone.0240576

**Published:** 2020-10-08

**Authors:** The PLOS ONE Editors

After this article [1] was published, concerns were raised about results reported in Figs 1 and 6.

Specifically:

- The following panels in Fig 1A appear to report overlapping data:
  ○ 37 and 39 degree panels for HepG2
  ○ 37 and 39 degree panels for Huh7
- Similarities were noted between Tubulin data reported in Fig 1B (HepG2 panel, lanes 2–5) and Fig 6C (lanes 1–4). The similar lanes represent samples exposed to different experimental conditions.

The corresponding authors commented that the invasion results for HepG2 and Huh7 cells were highly similar in the 37 and 39 degree groups. The original data underlying the results reported in Fig 1A are no longer available.

The authors provided available image data to support the results reported in Figs 1B and 6C, but these data did not resolve the concerns. The MMP-2, MMP-9, and Tubulin data reported in Fig 6C appear to match image data provided in support of Fig 1B, albeit with different blot and lane labels, suggesting that all three panels of Fig 6 may have reported the wrong data. None of the image data provided appear to match the results reported in Fig 1B. The authors commented that no additional western blot data are available currently.

In light of the unresolved concerns, the *PLOS ONE* Editors retract this article.

LY, JZ and WL agreed with retraction and apologize for the issues with the published article. The other authors either could not be reached or did not respond directly.
